# Supplementary material for: Synthesis of a Two-Dimensional Molybdenum Disulfide Nanosheet and Ultrasensitive Trapping of Staphylococcus Aureus for Enhanced Photothermal and Antibacterial Wound-Healing Therapy
Source: Nanomaterials (Basel). 2022 May 30;12(11):1865. doi: 10.3390/nano12111865 (PMC9182539; doi:10.3390/nano12111865)
Supplement: Supplementary file 1 [file nanomaterials-12-01865-s001.zip › nanomaterials-1716866-supplementary.pdf]

*Supporting Information*

# Synthesis of a Two-Dimensional Molybdenum Disulfide Nanosheet and Ultrasensitive Trapping of *Staphylococcus Aureus* for Enhanced Photothermal and Antibacterial Wound-Healing Therapy

**Weiwei Zhang [1,†], Zhao Kuang [1,†], Ping Song [1], Wanzhen Li [1], Lin Gui [2], Chuchu Tang [1], Yugui Tao [1,*], Fei Ge [1,*] and Longbao Zhu [1,*]**

[1] School of Biological and Food Engineering, Anhui Polytechnic University, Wuhu 241000, China; zwwjcf0908@163.com (W.Z.); kz827478890@163.com (Z.K.); songping1987@foxmail.com (P.S.); liwanzhen129@126.com (W.L.); tangcc2333@126.com (C.T.);

[2] Department of Microbiology and Immunology, Wannan Medical College, Wuhu 241002, China; guilin729@126.com

* Correspondence: swgctaoyg@126.com (Y.T.); gerrylin@126.com (F.G.); lbzhu2008@126.com (L.Z.)

† These authors contributed equally to this work.

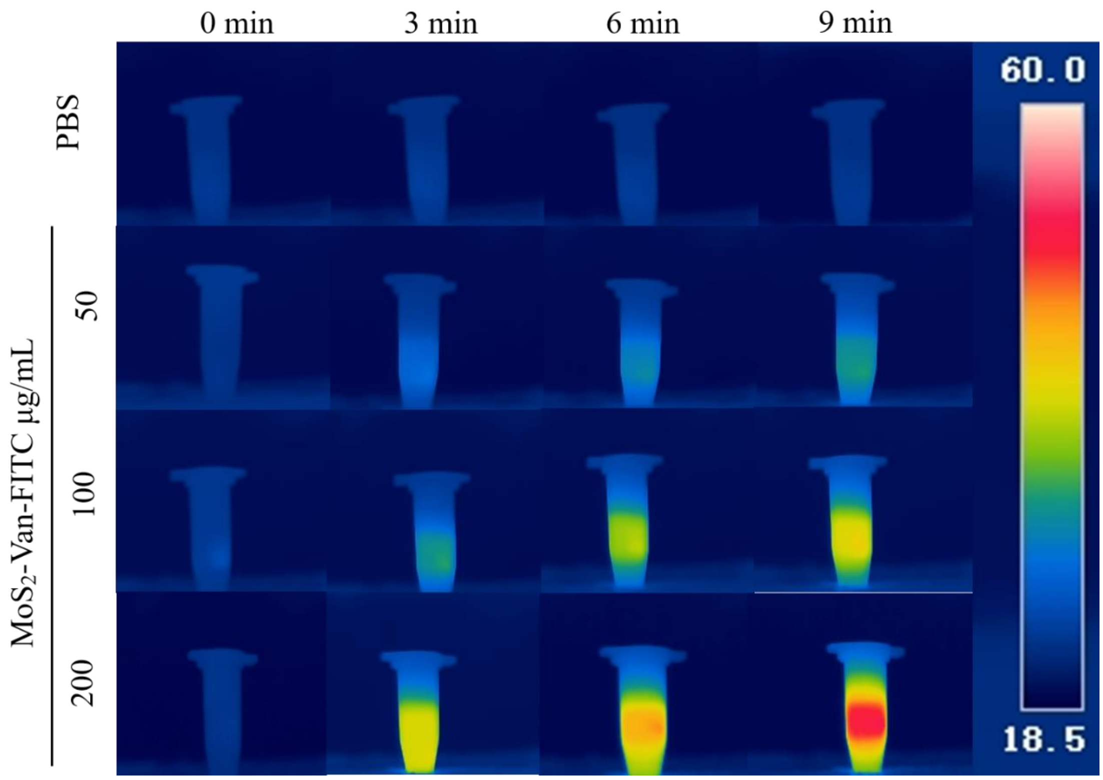


**Figure S1.** The thermal imaging images of $MoS_2$-Van-FITC@CS at different concentrations (50, 100 and 200 μg/mL) and NIR irradiation (808 nm; 2 W/cm$^2$). PBS served as a control.

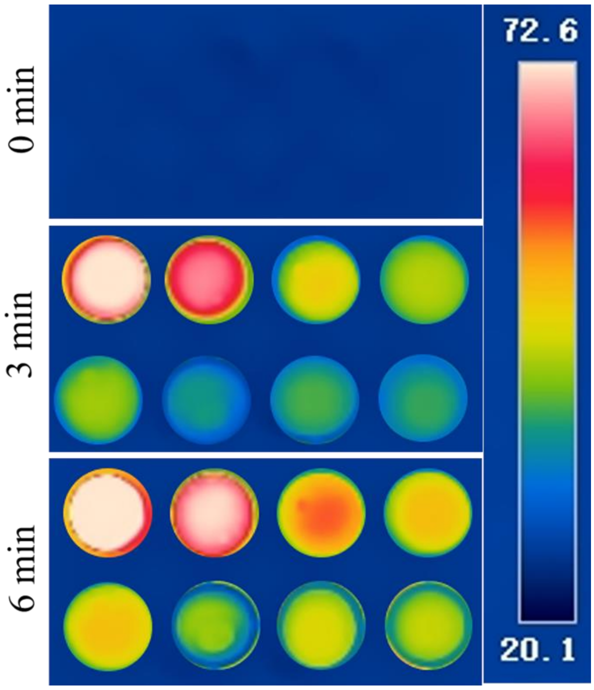


**Figure S2.** Thermal images of the test in MIC.
